# Supplementary material for: Accelerometry-assessed sleep duration and timing in late childhood and adolescence in Scottish schoolchildren: A feasibility study
Source: PLoS One. 2020 Dec 1;15(12):e0242080. doi: 10.1371/journal.pone.0242080 (PMC7707491; doi:10.1371/journal.pone.0242080)
Supplement: S1 Table — (DOCX) [file pone.0242080.s001.docx]

**S1 Table. Comparison of sample characteristics between pupils from low and high deprivation schools (SIMD)**

| Measure; *M* (SD) | High  (n = 24) | Low  (n = 37) | t/χ^2^ | p |
| --- | --- | --- | --- | --- |
| Datasets excluded, N (% of sample with accelerometry) | 7 (22.58) | 1 (2.63) | 6.63 | **0.010** |
| Age* | 11.88 (1.39) | 11.57 (0.55) | 1.21 | 0.232 |
| Secondary school, N (%) | 7 (29.17) | 24 (64.86) | 7.42 | **0.006** |
| Female*, N (%) | 13 (54.17) | 26 (70.27) | 1.64 | 0.441 |
| *missing,* N (%) | 1 (4.17) | 1 (2.70) |  |  |
| Number valid weekdays | 8.08 (1.69) | 8.54 (1.17) | 1.25 | 0.217 |
| Number of valid weekend days | 3.58 (0.83) | 3.63 (0.86) | 0.17 | 0.864 |
| Number of nights | 12.50 (2.43) | 13.19 (1.71) | 1.30 | 0.199 |
| Wore actigraph on dominant wrist, N (%) | 16 (66.67) | 18 (48.65) | 4.07 | 0.131 |
| *missing,* N (%) | 1 (4.17) | 0 (0) |  |  |
| **Accelerometry-derived activity measures** |  |  |  |  |
| Activity (ENMO) during least active 5 hours (L5) | 2.82 (0.78) | 3.07 (0.77) | 1.22 | 0.229 |
| Activity (ENMO) during most active 10 hours (M10) | 70.72 (22.02) | 61.70 (18.73) | 1.71 | 0.092 |
| **Accelerometry-derived sleep measures** |  |  |  |  |
| SPT-window duration | 8.55 (0.76) | 8.64 (0.70) | 0.48 | 0.636 |
| Sleep duration | 7.48 (0.79) | 7.49 (0.66) | 0.04 | 0.966 |
| Sleep efficiency | 0.88 (0.04) | 0.87 (0.03) | 1.32 | 0.193 |
| Sleep onset – weekdays | 22:36 (57.0) | 22:38 (47.4) | 0.13 | 0.901 |
| Sleep onset – weekends | 23:31 (78.6) | 23:29 (67.2) | 0.06 | 0.954 |
| Wake time – weekdays | 07:01 (52.2) | 07:04 (27.6) | 0.35 | 0.727 |
| Wake time - weekends | 08:07 (82.2) | 08:36 (64.8) | 1.55 | 0.127 |
| Meets sleep duration recommendations, N (%) | 0 (0) | 1 (2.70) | 0.66 | 0.417 |
| SPT-window meets recommended sleep duration, N (%) | 10 (41.67) | 11 (29.73) | 0.92 | 0.338 |
| **Accelerometry-derived circadian measures** |  |  |  |  |
| Relative amplitude | 0.92 (0.02) | 0.90 (0.03) | 2.44 | **0.018** |
| Social jetlag (hours) | 1.01 (0.92) | 1.20 (0.79) | 0.86 | 0.394 |
| **Questionnaires** | | | | |
| Total sleep score | 33.54 (9.66) | 38.38 (8.40) | 2.07 | **0.043** |
| Falling Asleep & Reinitiating Sleep | 18.38 (6.86) | 21.54 (5.72) | 1.95 | 0.056 |
| Returning to Wakefulness | 5.42 (2.48) | 5.49 (2.38) | 0.11 | 0.913 |
| Going to Bed | 9.75 (3.43) | 11.35 (2.85) | 1.98 | 0.053 |
| Mood and Feelings score | 5.67 (5.12) | 3.89 (3.39) | 1.63 | 0.108 |
| **Subjective sleep/circadian measures** | | | | |
| Subjective sleep onset – weekdays | 22:35 (58.2) | 21:49 (34.8) | 3.82 | **<0.001** |
| Subjective wake time - weekdays | 06:56 (41.8) | 07:14 (27.0) | 1.99 | 0.051 |
| Subjective sleep onset – weekends | 00:24 (91.8) | 22:45 (62.4) | 5.04 | **<0.001** |
| Subjective wake time - weekends | 09:19 (100.2) | 09:14 (76.8) | 0.18 | 0.856 |
| Subjective sleep duration – weekdays | 8.35 (1.39) | 9.41 (0.75) | 3.83 | **<0.001** |
| Subjective sleep duration - weekends | 8.92 (1.87) | 10.50 (1.37) | 3.80 | **<0.001** |
| Subjective social jetlag | 2.09 (1.24) | 1.47 (0.83) | 2.37 | **0.021** |
| **Feedback questions** |  |  |  |  |
| Problems with questionnaires? N (%) | 1 (4.17) | 0 (0) | 2.84 | 0.242 |
| Problems with actigraph? N (%) | 14 (58.33) | 18 (48.65) | 1.06 | 0.588 |

* Age and sex are estimated, see *Participants and Procedure*. For sleep onset and wake times, values are time in 24hr clock (SD in minutes). ENMO = Euclidean Norm Minus One; SIMD = Scottish Index of Multiple Deprivation; SPT = Sleep Period Time.
